# Sinonasal presentations of Rosai–Dorfman disease: a scoping review

**DOI:** 10.1017/S0022215125103046

**Published:** 2025-10

**Authors:** Kwasi Ansere Ofori, Ryan A. Rimmer

**Affiliations:** 1 School of Public Health, Yale University, New Haven, CT, USA; 2 School of Medicine - Division of Otolaryngology, Yale University, New Haven, CT, USA

**Keywords:** histiocytosis, sinus, nasal cavity, paranasal sinuses, nasal obstruction, epistaxis, immunohistochemistry, tomography, X-ray computed, positron emission tomography computed tomography

## Abstract

**Objectives:**

Rosai–Dorfman disease is a rare histiocytic disorder typically presenting with cervical lymphadenopathy. Sinonasal involvement is uncommon and presents diagnostic and therapeutic challenges. This scoping review synthesises literature on the clinical presentation, diagnosis, management and outcomes of sinonasal Rosai–Dorfman disease.

**Method:**

We systematically searched PubMed, Scopus and Embase. Articles were screened using Endnote. Studies reporting sinonasal Rosai–Dorfman disease were included. The review followed Preferred Reporting Items for Systematic Reviews and Meta-Analyses-ScR (Scoping Review) guidelines.

**Results:**

Thirty studies comprising 36 patients were included. Common symptoms were nasal obstruction (80.6 per cent) and epistaxis (41.7 per cent). Computed tomography (75 per cent) and magnetic resonance imaging (36.1 per cent) were primary imaging modalities. Histopathology showed emperipolesis (66.7 per cent), S-100 (69.4 per cent) and CD68 (47.2 per cent) positivity. Management was mainly surgical (72.2 per cent), with corticosteroids (44.4 per cent), radiotherapy (5.6 per cent) and chemotherapy (5.6 per cent) used less frequently. Outcomes included complete resolution (38.9 per cent), stable disease (38.9 per cent) and recurrence (16.7 per cent).

**Conclusion:**

Diagnosis relies on histopathology and imaging. Surgical procedures, often with corticosteroids, remain the primary treatment. Future research should guide diagnostic and treatment protocols.

## Introduction

Rosai–Dorfman disease is a rare histiocytic disorder first described in 1965 by Pierre Louis Destombes.^1^ Histiocytoses are rare conditions involving the accumulation of cells such as macrophages and dendritic cells.^2^ According to the 2016 revised histiocytosis classification, Rosai–Dorfman disease is under the ‘R’ group, encompassing its classical, sporadic and extranodal forms, and including variants associated with immune-mediated conditions such as systemic lupus erythematosus and neoplasia.^2^ This classification reflects the clinical heterogeneity of Rosai–Dorfman disease and distinguishes it by its hallmark histiocytes with emperipolesis and S-100 positivity without CD1a expression.^2^

Rosai–Dorfman disease affects approximately 1 in 200 000 individuals, with about 100 new cases reported annually in the USA.^3^ It frequently presents as bilateral cervical lymphadenopathy in children and adults.^4^ Classical sporadic Rosai–Dorfman disease affects the lymph nodes and is more common in children and young adults, especially males of African descent.^4^ Painless cervical lymphadenopathy, which is present in 80 per cent of cases, is often accompanied by systemic symptoms, including fever, night sweats, fatigue and weight loss.^2^^,^^5^ Other lymph node sites may be involved.^2^

Rosai–Dorfman disease clinical behaviour encompasses moderate, disseminated and occasionally life-threatening forms.^2^ Mild to moderate presentations often include isolated, painless cervical lymphadenopathy with or without systemic symptoms such as fever and night sweats. Disseminated disease may involve multiple extranodal sites, including the skin, orbit or paranasal sinuses.^2^ Life-threatening cases may result from mass effect on the airway, brain or spinal cord.^6^^,^^7^ Extranodal disease occurs in about 43 per cent of cases, affecting sites such as the skin, central nervous system and orbit.^1^ Sinonasal involvement is rare, present in 11 per cent of cases and can be diagnostically challenging because of its similarity to other conditions.^8^^,^^9^ Rosai–Dorfman disease-like histiocytes identified in chronic rhinosinusitis have prompted debate over classification.^9^ Our scoping review aimed to synthesise current knowledge on the presentation, diagnosis and management of sinonasal Rosai–Dorfman disease, identifying gaps to inform future research and improve clinical practice.

## Materials and methods

This scoping review adhered to the followed Preferred Reporting Items for Systematic Reviews and Meta-Analyses (PRISMA)-ScR guidelines and involved a structured search of three databases, PubMed, Scopus and Embase, to gather relevant literature on sinonasal Rosai–Dorfman disease. These databases were chosen for their ability to index rare diseases and offer comprehensive coverage of biomedical and multidisciplinary literature.

The search targeted articles on sinonasal Rosai–Dorfman disease with the keywords ‘Rosai–Dorfman disease’, ‘sinonasal’, ‘sinus’, ‘nasal cavity’, ‘paranasal sinuses’ and ‘histiocytosis’ using Boolean operators (AND, OR) to refine the results. The complete search strategy can be found in Appendix 1.

Titles and abstracts were reviewed to identify potentially relevant studies and full-text reviews were conducted for articles meeting the inclusion criteria. Selected papers were then imported into EndNote for reference management and deduplication. Two reviewers independently screened and assessed the articles, and discrepancies were resolved through discussion to minimise bias.

Studies were included if the full text could be reviewed in English, reported a case or case series of sinonasal Rosai–Dorfman disease and included data on clinical features, diagnostic approaches or treatment strategies. Studies were excluded if they focused solely on nodal or non-sinonasal Rosai–Dorfman disease, were reviews, editorials or conference abstracts without detailed case data, or were non-English publications.

We created a table to extract key information from each study, including patient demographics, clinical presentation, diagnostic tools, treatment modalities and outcomes. Findings were synthesised descriptively to identify patterns in the presentation, diagnosis and treatment of sinonasal Rosai–Dorfman disease. Where appropriate, summary statistics and visual aids such as tables and figures were used to highlight trends and variations.

The Joanna Briggs Institute Critical Appraisal Tool was used to assess study quality in line with PRISMA-ScR, supporting the review’s exploratory aims.

## Results and analysis

A total of 154 articles were screened, with 30 studies (25 case reports and 5 case series) meeting inclusion criteria, comprising 36 patients with sinonasal Rosai–Dorfman disease.^5^^,^^8^^,^^10^^–^^37^ Patients’ ages ranged from 6 to 78 years (mean, 40.8**[Qwas.6]** ± 19.4 years), with a slight male preponderance (52.8 per cent) (Table 1). Most cases were reported in adults and seven paediatric cases were identified. Studies originated from 16 countries, with the USA (7 studies), India (6 studies) and Hong Kong (5 studies) contributing the most.

**Table 1.** Patient characteristics

Characteristics	Value (*n* (%))
Total patients	36
Male	19 (52.8)
Female	17 (47.2)
Age (mean ± SD; years)	40.8 ± 19.4
Age range (years)	6−78

SD = standard deviation.

Nasal obstruction was the most common symptom, reported in 29 cases (80.6 per cent), followed by epistaxis (15 cases, 41.7 per cent), headache (7 cases, 19.4 per cent), sleep-disordered breathing symptoms (6 cases, 16.7 per cent), dyspnoea (5 cases, 13.9 per cent) and visual disturbances (4 cases, 11.1 per cent) (Table 2). Cervical lymphadenopathy was present in 14 cases (38.9 per cent).

**Table 2.** Patient clinical presentations/symptoms

Symptom	Number of cases (*N* = 36)	%
Nasal obstruction	29	80.6
Epistaxis	15	41.7
Headache	7	19.4
Sleep-disordered breathing symptoms	6	16.7
Dyspnoea	5	13.9
Visual disturbances	4	11.1

All patients underwent diagnostic evaluation using multiple tools, with histopathological analysis typically based on sinonasal tissue and, in some cases, fine needle aspiration cytology (FNAC) of cervical lymph nodes. Immunohistochemical testing commonly included S-100, CD68 and immunoglobulin levels, while lymphocyte markers such as CD1a, CD3 and CD20 were used to exclude hematologic malignancies. Tissue sampling methods ranged from in-clinic biopsy to endoscopic sinus surgery and craniotomy. Emperipolesis is a non-destructive process in which a viable cell transiently resides within another living cell without structural or functional harm to either.^38^ It is a hallmark feature of Rosai–Dorfman disease, typically accompanied by S-100 and CD68 positivity, key diagnostic features reported in 66.7, 69.4 and 47.2 per cent of cases, respectively.

All cases with available immunohistochemical data were positive for at least one of S-100 or CD68. Elevated immunoglobulin levels (IgG, IgA or IgG4) were observed in 6 cases (16.7 per cent). CD1a, CD3, CD20 and CD138 were each positive in one case, while all other lymphoma markers were negative. Erythrocyte sedimentation rate was documented in 11 cases (30.6 per cent), with only two normal results.

**Figure 1.**
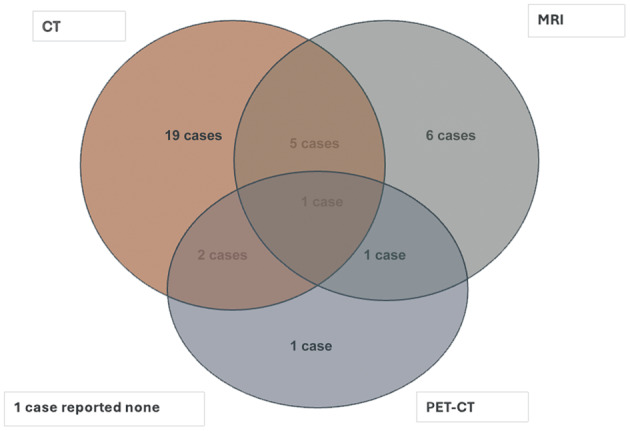
Venn diagram illustrating the use and overlap of imaging modalities in the diagnosis of sinonasal Rosai–Dorfman disease. CT = computed tomography; MRI = magnetic resonance imaging; PET-CT = positron emission tomography.

Computed tomography (CT) scans were used to evaluate disease in 27 cases (75 per cent) and magnetic resonance imaging (MRI) in 13 cases (36.1 per cent), with 5 patients receiving both. Positron emission tomography CT (PET-CT) was used in 5 cases (13.9 per cent) and 1 case utilised all three modalities. Imaging was performed in all but one case. Figure 1 illustrates the distribution of imaging modalities in our study.

**Figure 2.**
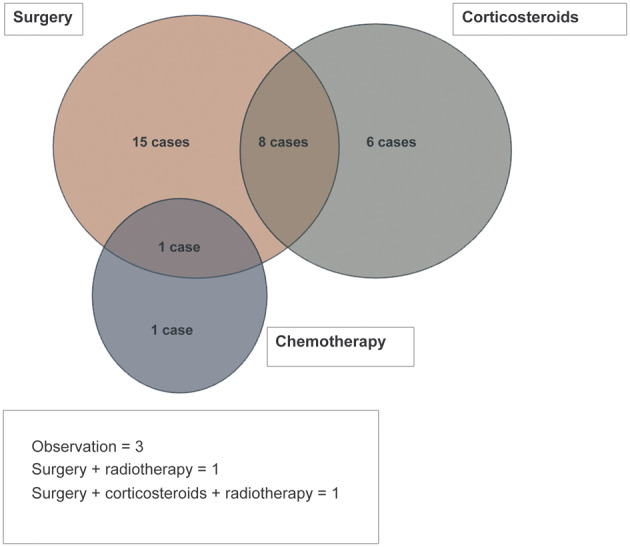
Venn diagram showing the overlap of treatment strategies used across sinonasal Rosai–Dorfman disease cases, including surgery, corticosteroids, chemotherapy and radiotherapy.

Management involved multidisciplinary teams, including otolaryngology, pathology, radiology, medical oncology and radiation oncology. Treatment strategies varied by disease extent and severity. Surgical intervention was the most common approach, performed in 26 cases (72.2 per cent), using techniques such as endoscopic sinus surgery, carbon dioxide (CO_2_) laser vaporisation and craniotomy. Corticosteroids were used in 16 cases (44.4 per cent), including 8 as adjuncts to surgery. Radiotherapy was used in two cases and chemotherapy was administered in two cases with systemic disease. Observation without active treatment was selected in 3 cases (8.3 per cent), some of which showed spontaneous regression. Figure 2 shows the distribution of treatment modalities.

Recurrence was reported in six cases, with varied timelines and management strategies. One patient developed pericardial involvement three years after sinonasal surgery, responding to cladribine after methotrexate failure.^19^ Another patient experienced recurrence within six months after surgical resection of extensive sinonasal and orbital disease and was subsequently lost to follow up.^24^ In another case, a subglottic mass recurred following CO_2_ laser debulking, with persistent sinonasal lesions and lymphadenopathy.^29^ A patient with coexisting rhinoscleroma had surgical excision via the Caldwell–Luc approach but experienced minimal sinonasal recurrence, which was effectively managed with endoscopic sinus surgery.^29^ A 72-year-old woman required repeat laser sessions within 3 months, achieving disease control by 10 months.^11^ Finally, a 54-year-old man experienced symptom recurrence 3 years post-surgery but achieved sustained remission following systemic steroid therapy, with no recurrence during 3-year follow up.^36^

Among the reported cases, 14 patients (38.9 per cent) achieved complete resolution, while another 14 (38.9 per cent) had stable disease, including 1 with treatment-limiting complications due to immunosuppression. Recurrence occurred in 6 cases (16.7 per cent), often within 6 months post-treatment. Two patients (5.6 per cent) lacked follow-up data. Follow-up durations ranged from six weeks to five years.

## Discussion

Rosai–Dorfman disease is considered a benign histiocytic disease typically presenting with massive, bilateral cervical lymphadenopathy, but sinonasal manifestations are uncommon.^8^^,^^23^ Diagnosis at index presentation appears to be a challenge, often attributed to the presence of nonspecific sinonasal complaints, rarity and histological similarities to other diseases.^39^^,^^40^

Cervical lymphadenopathy was present in over a third of sinonasal Rosai–Dorfman disease cases and is frequently misdiagnosed as infection, granulomatous disease or malignancy.^39^ Reports include a 45-year-old woman initially diagnosed with odontogenic infection, a 33-year-old man misdiagnosed with tuberculosis and fungal sinusitis, and a 45-year-old man misdiagnosed with lymphoma, all later confirmed to have Rosai–Dorfman disease.^41^^–^^43^ Rosai–Dorfman disease can be differentiated from Langerhans cell histiocytosis by emperipolesis and its characteristic immunophenotype, CD68^+^/S-100^+^/CD1a^−^.^2^^,^^44^^,^^45^ Rarely, the disease may extend intracranially and mimic meningioma, but its hallmark histologic features aid differentiation, underscoring the need for heightened diagnostic awareness.^35^^,^^46^^,^^47^

Although FNAC may suggest Rosai–Dorfman disease, it is often inconclusive and can be confounded by benign mimics, thus definitive diagnosis typically requires tissue biopsy with detailed histopathological and immunohistochemical evaluation.^29^^,^^39^^,^^48^^,^^49^

Of the two cases tested for Epstein–Barr virus (EBV), one was positive. Rosai–Dorfman disease is thought to involve immune dysregulation triggered by an infectious agent, specifically the EBV.^39^ Other implicated disease-causing pathogens include human herpesvirus (HHV)-6, Varicella zoster virus, Cytomegalovirus, *Klebsiella* spp. and *Brucella* spp.^11^^,^^50^ However, the aetiology of Rosai–Dorfman disease remains unclear, and the role of EBV is not well-established, hence future research should systematically evaluate EBV status in Rosai–Dorfman disease patients to determine whether EBV-driven immune responses contribute to disease pathogenesis.^39^^,^^50^

Rosai–Dorfman disease is often multifocal, and diagnosis in one area should prompt suspicion of multi-system involvement.^51^ Although ultrasonography is recommended as the initial imaging modality for children with neck masses, it may be insufficient in suspected Rosai–Dorfman disease cases.^52^ A two-year-old girl with cervical lymphadenopathy had normal ultrasound findings, but CT imaging revealed a sinonasal mass with sphenoid sinus erosion and orbital extension, highlighting the critical role of comprehensive imaging in patient evaluation.^39^^,^^53^

Given Rosai–Dorfman disease’s potential for systemic involvement, PET-CT is increasingly utilised to detect subclinical disease, particularly in patients with otherwise localised symptoms. Studies suggest PET-CT provides additional diagnostic information in 30 per cent of cases where CT or MRI is inconclusive, leading to management modifications in up to 41 per cent of patients.^34^^,^^54^ In one sinonasal Rosai–Dorfman disease case, PET-CT detected skeletal involvement missed by prior imaging, prompting a change in treatment.^34^ In another, a 42-year old woman with longstanding extranodal Rosai–Dorfman disease underwent PET-CT monitoring over nearly a decade, which revealed recurrent Fluorodeoxyglucose (FDG)-avid lesions and guided treatment with corticosteroids, radiotherapy and 2-chlorodeoxyadenosine, resulting in partial responses.^55^ Routine PET-CT should be considered in cases with suspected systemic involvement or unclear disease extent on conventional imaging.^54^

Treatment for Rosai–Dorfman disease is primarily indicated for symptomatic cases or those involving critical organ dysfunction.^40^^,^^56^ While some cases resolve spontaneously, up to 70 per cent may persist.^35^^,^^57^ Localised symptomatic disease is generally treated with surgery or radiotherapy, while asymptomatic cases are monitored.^56^^,^^57^

In our review, surgery was the most common treatment, particularly for resectable sinonasal disease. A systematic review of paediatric otorhinolaryngologic Rosai–Dorfman disease found surgical excision to be the most common and effective treatment, with the lowest recurrence rates.^47^ However, several studies emphasise the value of combining medical and surgical therapies, and the importance of individualised treatment strategies.^57^ If complete resection is not feasible, adjuvant chemotherapy or radiotherapy may support disease control.^13^ Laser excision is a minimally invasive option for nasal lesions, while corticosteroids and endoscopic surgery are useful in recurrent or compressive cases. However, the optimal surgical approach remains undefined.^11^

Corticosteroids were suggested as the first-line treatment in several cases, but with limited efficacy in severe disease.^57^ Studies suggest treatment with corticosteroids results in transient decrease in lymph node size but rebound frequently occurs after treatment.^13^ In other studies, however, corticosteroids were shown to effectively reduce fever and lymphadenopathy in Rosai–Dorfman disease, with symptom resolution reported within a range of 5 days to 6 months.^13^ These highlight the variability in responses, with some patients requiring prolonged therapy or experiencing relapse.^58^ While corticosteroids are valuable for symptom control, long-term management may necessitate additional therapeutic approaches.

A patient in our review received methotrexate, vinblastine and 6-mercaptopurine (MP), indicating a potential role for immunosuppressive agents in select cases.^10^ Additionally, a 64-year-old man with refractory, disseminated Rosai–Dorfman disease reported complete remission following treatment with siltuximab, an anti-interleukin (IL)-6 monoclonal antibody, with minimal adverse effects.^56^ The role of IL-6 in Rosai–Dorfman disease pathophysiology suggests that cytokine dysregulation may contribute to disease progression.^56^ While the lack of IL-6 measurement is a limitation, the favourable clinical response supports the need for further investigation. A separate review of seven Rosai–Dorfman disease patients, five with prior treatment failures, found that thalidomide led to clinical improvement in five cases and minimal response in two.^59^ Thalidomide, an oral immunomodulatory agent, exerts anti-inflammatory effects through cytokine modulation, tumour necrosis factor-α inhibition and T-helper cell regulation.^59^^,^^60^ However, its use is limited by significant adverse effects, including hypothyroidism, deep vein thrombosis and teratogenicity.^60^

Cases of widespread systemic Rosai–Dorfman disease often require multidisciplinary management involving surgical specialists, oncology, rheumatology and immunology. Radiotherapy may be considered for refractory or isolated lesions, but its overall effectiveness has been limited in prior reports.^61^

Rosai–Dorfman disease has an unpredictable disease course, with approximately 50 per cent of cases resolving spontaneously, 33 per cent showing residual asymptomatic lymphadenopathy and 17 per cent exhibiting persistent disease.^24^ Clinical assessment and imaging should be performed every 6 to 12 months, particularly in patients with prior recurrence or incomplete resection. Surgical cases may require earlier imaging, often within three to six months, to assess for residual disease.

Long-term disease monitoring is essential, as highlighted by a case of sinonasal Rosai–Dorfman disease diagnosed in the 1990s that recurred over a decade later with symptomatic tracheobronchial obstruction requiring mechanical resection.^62^ A clinical study of 10 sinonasal Rosai–Dorfman disease cases over 7 years reported a high recurrence rate, reinforcing the importance of extended surveillance.^63^

Asymptomatic or mild cases of Rosai–Dorfman disease may be managed with observation and routine follow-up, given the possibility of spontaneous resolution. For symptomatic, localised sinonasal disease, endoscopic surgery is often the preferred initial treatment. Corticosteroids may be useful in recurrence or diffuse inflammation, while refractory disease may require adjunctive therapies such as radiotherapy, chemotherapy or targeted agents like siltuximab, although evidence remains limited. In multifocal or systemic disease, PET-CT should guide treatment decisions, and a multidisciplinary approach involving surgery, oncology and immunology is advised. CT remains the best imaging modality for evaluating bony anatomy and disease extent.^64^

For post-surgical surveillance, endoscopy and imaging (MRI, CT or PET-CT) are advised, with MRI showing the highest positive predictive value.^65^ According to National Comprehensive Cancer Network (NCCN) guidelines for sinonasal tumours, 80–90 per cent of recurrences occur within the first 2–4 years, so surveillance typically includes 4–12 visits in the first year, 3–6 in the second, 2–3 annually from years 3 to 5 and then either yearly follow up or discontinuation.^66^ Studies suggest that up to one-third of follow-up visits may be unnecessary, highlighting the need for individualised surveillance.^66^

This review consolidates current knowledge on sinonasal Rosai–Dorfman disease, emphasising the diagnostic and therapeutic challenges associated with this rare condition. Future research should aim to refine diagnostic criteria, establish standardised treatment protocols and identify markers predictive of disease progression and recurrence.

Our findings are limited by the rarity of sinonasal Rosai–Dorfman disease, with most data drawn from retrospective case reports and small series. The absence of uniform diagnostic and treatment guidelines, along with inconsistent reporting of key clinical details, such as immunohistochemical findings, EBV status, follow-up duration and recurrence, hinders the ability to draw definitive conclusions. Only two cases included EBV testing, leaving its role in disease pathogenesis unclear. Additionally, long-term outcomes were often inadequately documented, making the true recurrence risk difficult to assess.

Larger, prospective studies with standardised reporting and extended follow up are essential to improve the clinical decision-making and long-term management of sinonasal Rosai–Dorfman disease.

- Sinonasal Rosai–Dorfman disease is rare and presents with non-specific symptoms that complicate timely diagnosis
- Surgical intervention, occasionally combined with corticosteroids, remains the primary treatment modality for symptomatic disease
- Histopathology showing emperipolesis, S-100⁺/CD68⁺ staining and imaging are essential for diagnosis
- PET-CT is increasingly valuable for assessing systemic involvement, tracking recurrence and treatment response
- Future research should focus on standardised diagnostic criteria, optimised therapies and long-term surveillance protocols

## Conclusion

Sinonasal Rosai–Dorfman disease is a rare disease that remains challenging to diagnose and manage because of its variable presentation and lack of standardised guidelines. Diagnosis often requires a combination of tissue analysis and immunohistochemistry. Treatment varies widely, with surgery being the most common approach, often combined with corticosteroids, radiation or chemotherapy. PET-CT has proven useful in tracking disease progression and detecting recurrence, suggesting its potential role in long-term follow up. With the risk of recurrence and the absence of clear guidelines, future research should focus on refining diagnostic criteria, optimising treatment plans and developing structured follow-up protocols. Collaboration among specialists is essential to improving patient care and expanding our understanding of this uncommon but important disease.

## Appendix 1. Search strategies

### Pubmed

(‘Rosai-Dorfman disease’[MeSH Terms] OR ‘Rosai-Dorfman disease’[Title/Abstract] OR ‘Sinus histiocytosis with massive lymphadenopathy’[Title/Abstract] OR ‘SHML’[Title/Abstract]) AND (‘Sinonasal’[Title/Abstract] OR ‘Head and Neck’[MeSH Terms] OR ‘Nasal cavity’[MeSH Terms] OR ‘Paranasal sinuses’[MeSH Terms] OR ‘Ethmoid sinus’[Title/Abstract] OR ‘Maxillary sinus’[Title/Abstract] OR ‘Frontal sinus’[Title/Abstract] OR ‘Sphenoid sinus’[Title/Abstract] OR ‘Nasal mass’[Title/Abstract] OR ‘Nasal discharge’[Title/Abstract] OR ‘Nasal obstruction’[Title/Abstract] OR ‘Rhinosinusitis’[Title/Abstract] OR ‘Sinus tumor’[Title/Abstract])

### Embase

(‘rosai-dorfman disease’ OR ‘sinus histiocytosis with massive lymphadenopathy’) AND (‘sinonasal’ OR ‘nasal cavity’ OR ‘paranasal sinus’ OR ‘frontal sinus’ OR ‘maxillary sinus’ OR ‘sphenoid sinus’ OR ‘ethmoid sinus’ OR ‘nasal obstruction’ OR ‘rhinosinusitis’) NOT (‘skin’ OR ‘orbital’ OR ‘bone’ OR ‘CNS’ OR ‘lymph nodes’)

### Scopus (Advanced search)

TITLE-ABS(‘Rosai-Dorfman disease’ OR ‘Sinus histiocytosis with massive lymphadenopathy’ OR ‘SHML’) AND TITLE-ABS(‘sinonasal’ OR ‘nasal cavity’ OR ‘paranasal sinuses’ OR ‘ethmoid sinus’ OR ‘maxillary sinus’ OR ‘frontal sinus’ OR ‘sphenoid sinus’ OR ‘nasal mass’ OR ‘nasal obstruction’ OR ‘rhinosinusitis’)

*Date of most recent search: 8 February 2025.

## References

[1] Elbaz Younes I, Sokol L, Zhang L. Rosai–Dorfman disease between proliferation and neoplasia. *Cancers (Basel)* 2022;14:5271 36358690 10.3390/cancers14215271 PMC9654168

[2] Emile J-F, Abla O, Fraitag S, Horne A, Haroche J, Donadieu J, et al. Revised classification of histiocytoses and neoplasms of the macrophage-dendritic cell lineages. *Blood* 2016;127:2672–81 26966089 10.1182/blood-2016-01-690636 PMC5161007

[3] Werneck Rodrigues DO, Wolp Diniz R, Dentz LC, Costa MDA Lopes RH, Suassuna LF, et al. Case study: Rosai–Dorfman disease and its multifaceted aspects. *J Blood Med* 2024;15:123 38495774 10.2147/JBM.S436720 PMC10941986

[4] Bruce-Brand C, Schneider JW, Schubert P. Rosai–Dorfman disease: an overview. *J Clin Pathol* 2020;73:697–705 32591351 10.1136/jclinpath-2020-206733

[5] Dier KD, Lemmerling M, De Vos G. Nasal and nasopharyngeal Rosai–Dorfman disease. *J Belg Soc Radiol* 2022;106:86 36213369 10.5334/jbsr.2896 PMC9503955

[6] Dran G, Rasendrarijao D, Vandenbos F, Paquis P. Rosai–Dorfman disease causing spinal cord compression: case report. *Neurosurgery* 2008;62:E977-8 18496169 10.1227/01.neu.0000318189.56277.b8

[7] Leonhardt LP, Yamagata H, Harcha J, Calvo A. Achievement of clinical remission in life-threatening Rosai–Dorfman disease with cladribine. *Cureus* 2024;16:e74576 39735017 10.7759/cureus.74576 PMC11673318

[8] Dhakal S, Katwal S, Ghimire A, Bhusal A, Yogi TN. Uncommon presentation of Rosai–Dorfman disease: nasal and nasopharyngeal involvement. *Radiol Case Rep* 2024;19:956–60 38204935 10.1016/j.radcr.2023.11.055 PMC10776908

[9] Rooper LM, White MJ, Duffield AS, Gagan J, London Jr NR Montgomery EA, et al. Limited sinonasal Rosai–Dorfman disease presenting as chronic sinusitis. *Histopathology* 2022;81:99–107 35426462 10.1111/his.14664 PMC9324200

[10] Cachay LCA. Patient with Rosai–Dorfman–Destombes disease: 18F-FDG-PET/CT scan as a diagnostic tool. *Acta Medica Peruana* 2023;39:396–8

[11] Belcadhi M, Bellakhdhar M, Sriha B, Bouzouita K. Rosai–Dorfman disease of the nasal cavities: a CO_2_ laser excision. *Am J Rhinol Allergy* 2010;24:91–3 20109334 10.2500/ajra.2010.24.3387

[12] Bhat SP, Prasad K, Satheesh B, Bhandary K, Baikunje S, Bhat V. Metachronous nodal and nasal Rosai–Dorfman disease presenting with chronic renal failure. *Clin Rhinol An Int J* 6:139–43

[13] Burgos-Sosa E, Jose de Jesus Julian Mendoza, Chavez-Macias L, Ichazo-Castellanos JP, Rodriguez MAG, Garcia-Guzman B, et al. Rosai–Dorfman sphenoorbital histiocytosis with intraparenchymal invasion: do we have to consider this skull base pathology as a malignant disease? *Surg Neurol Int* 2024;15:337 39373001 10.25259/SNI_405_2024 PMC11450868

[14] Cardoso CL, Damante JH, Da Silva Santos PS, De Assis Taveira LA, Da Silva Ramos LMP, Pigatti FM, et al. Rosai–Dorfman disease with widespread oral-maxillofacial manifestations: a case report. *J Oral Maxillofac Surg* 2012;70:2600–4 22330332 10.1016/j.joms.2011.12.015

[15] Dodson KM, Powers CN, Reiter ER. Rosai Dorfman disease presenting as synchronous nasal and intracranial masses. *Am J Otolaryngol* 2003;24:426–30 14608579 10.1016/s0196-0709(03)00090-5

[16] El-Banhawy OA, Farahat HG, El-Desoky I. Facial asymmetry with nasal and orbital involvement in a case of sinus histiocytosis with massive lymphadenopathy (Rosai–Dorfman disease). *Int J Pediatr Otorhinolaryngol* 2005;69:1141–5 16005357 10.1016/j.ijporl.2005.02.021

[17] Goodnight JW, Wang MB, Sercarz JA, Fu YS. Extranodal Rosai–Dorfman disease of the head and neck. *Laryngoscope* 1996;106:253–6 8614184 10.1097/00005537-199603000-00002

[18] Gupta L, Batra K, Motwani G. A rare case of Rosai–Dorfman disease of the paranasal sinuses. *Indian J Otolaryngol Head Neck Surg* 2005;57:352–4 23120220 10.1007/BF02907713 PMC3451433

[19] Haghighat Jahromi A, Goodman AM, Hoh CK. Rosai–Dorfman–Destombes (RDD) disease presenting as palindromic rheumatism. *BMC Med Imaging* 2021;21:1–5 33858362 10.1186/s12880-021-00596-2 PMC8050901

[20] Heaton C, Murr A, Jones KD, Naujokas A, Karlon W. Extranodal Rosai–Dorfman disease presenting as an intranasal mass. *Laryngoscope* 2011;**121**(Suppl 4):S75

[21] Houas J, Ghammam M, BelHadj-Miled H, Kedissa A, Bellakhdher M, Abdelkefi M. Destombes–Rosai–Dorfman disease: a case series of nodular and extra nodular involvement. *Int J Surg Case Rep* 2024;125:110589 39536678 10.1016/j.ijscr.2024.110589 PMC11605385

[22] Kelly WF, Bradey N, Scoones D. Rosai–Dorfman disease presenting as a pituitary tumour. *Clin Endocrinol (Oxf)* 1999;50:133–7 10341867 10.1046/j.1365-2265.1999.00461.x

[23] Khan AA, Siraj F, Rai D, Aggarwal S. Rosai–Dorfman disease of the paranasal sinuses and orbit. *Hematol Oncol Stem Cell Ther* 2011;4:94–6 21727770 10.5144/1658-3876.2011.94

[24] Shiran MS, Tan GC, Kenali MS, Sabariah AR, Pathmanathan R. Multifocal nodal and extranodal Rosai–Dorfman disease initially diagnosed as histiocytic lymphoma. *Malays J Pathol* 2008;30:63–5 19108414

[25] Walid MS, Grigorian AA. Ethmo-spheno-intracranial Rosai–Dorfman disease. *Indian J Cancer* 2010;47:80–1 20071801 10.4103/0019-509X.58870

[26] Wang J, Wang Y, Li G, Wang C, Yu G, Sun Y. Rosai–Dorfman disease originating from nasal septal mucosa and presenting with nasal dorsum collapse: a case report with literature review. *Ear Nose Throat J* 2024;103:NP604–9 35171732 10.1177/01455613221079500

[27] Warpe BM, More SV. Rosai–Dorfman disease: a rare clinico-pathological presentation. *Australas Med J* 2014;7:68–72 24611075 10.4066/AMJ.2014.1931 PMC3941579

[28] Zhong Z, Fu W, Sun Y, Sun Q, Bian L. Giant isolated transcranial Rosai–Dorfman disease with diffuse involvement of nasal and paranasal tissues: case report and literature review. *Br J Neurosurg* 2019;33:299–301 28670983 10.1080/02688697.2017.1344614

[29] Lai KL, Abdullah V, Ng KS, Fung NS, Van Hasselt CA. Rosai–Dorfman disease: presentation, diagnosis, and treatment. *Head Neck* 2013;35:E85–8 22083607 10.1002/hed.21930

[30] Natarajan K, Devarasetty A, Murali S, Senthilvadivu A, Sudhamaheswari, Kameswaran M. Sinonasal Rosai–Dorfman disease. *Clin Rhinol* 2014;7:142–6

[31] Lee M, Ryu KH, Baek HJ, Moon J Il, Yoon S, An HJ, et al. Rosai–Dorfman disease with infiltration of IgG4-bearing plasma cells presenting as laryngeal-nasal masses and cervical lymphadenopathy: a case report. *Medicine (Baltimore)* 2021;100:e25165 33761691 10.1097/MD.0000000000025165 PMC9281989

[32] Ku PKM, Tong MCF, Leung CY, Pak MW, Van Hasselt CA. Nasal manifestation of extranodal Rosai–Dorfman disease – diagnosis and management. *J Laryngol Otol* 1999;113:275–80 10435144 10.1017/s0022215100143786

[33] Kumari JO. Coexistence of rhinoscleroma with Rosai–Dorfman disease: is rhinoscleroma a cause of this disease? *J Laryngol Otol* 2012;126:630–2 22643208 10.1017/S0022215112000552

[34] Oui TJ, Zahedi FD, Husain S, Wan Hamizan AK. Rare extranodal manifestation of Rosai–Dorfman disease presenting as nasal obstruction and its management. *BMJ Case Reports* 2023;16:e251801 10.1136/bcr-2022-251801 PMC10335459 37407235

[35] Kim GG, Friedel ME, Eloy JA, Jyung RW, Liu JK. Extensive multifocal Rosai–Dorfman disease involving the central nervous system and paranasal sinuses. *Laryngoscope* 2011;**121**(Suppl 4):S234

[36] Hagemann M, Zbären P, Stauffer E, Caversaccio M. Nasal and paranasal sinus manifestation of Rosai–Dorfman disease. *Rhinology* 2005;43:229–32 16218519

[37] Ilie M, Guevara N, Castillo L, Hofman P. Polypoid intranasal mass caused by Rosai–Dorfman disease: a diagnostic pitfall. *J Laryngol Otol* 2010;124:345–8 19646302 10.1017/S0022215109990818

[38] Rastogi V, Sharma R, Misra SR, Yadav L, Sharma V. Emperipolesis – a review. *J Clin Diagn Res* 2014;8:ZM01 10.7860/JCDR/2014/10361.5299 PMC4316366 25654060

[39] Azari-Yaam A, Abdolsalehi MR, Vasei M, Safavi M, Mehdizadeh M. Rosai–Dorfman disease: a rare clinicopathological presentation and review of the literature. *Head Neck Pathol* 2021;15:352–60 32504287 10.1007/s12105-020-01183-7 PMC8010025

[40] Xu H, Zhang H, Li W, Zhang C, Wang H, Wang D. Nasal presentations of Rosai–Dorfman disease: clinical manifestation and treatment outcomes. *Ear Nose Throat J* 2023;102:NP1–8 36884341 10.1177/01455613231162226

[41] Chen Y, Ma W, Nie G, Li M, Cui Q. One case of Rosai–Dorfman disease misdiagnosed as facial inflammation. *Hua Xi Kou Qiang Yi Xue Za Zhi* 2024;42:671–4 39304512 10.7518/hxkq.2024.2023455 PMC11493861

[42] Abdela SG, Mengesha CA. Rosai–Dorfman disease mimicking gastrointestinal tuberculosis and fungal sinusitis: a case report. *Radiol Case Rep* 2022;17:4730–3 36212757 10.1016/j.radcr.2022.09.024 PMC9539622

[43] Su X, Zhang L. Orbital Rosai–Dorfman disease: a case report and literature review. *J Int Med Res* 2019;47:5891–5 31612761 10.1177/0300060519878086 PMC6862901

[44] Rastogi A, Jaisingh K, Rajurkar K, Saran RK, Singh M, Baindur S, et al. Coexistent Rosai Dorfman disease and Langerhans cell histiocytosis in an orbital mass: a case report. *Eur J Ophthalmol* 2023;33:NP60–5 10.1177/11206721221083727 35229680

[45] Kishore M, Gupta P, Ahuja A, Bhardwaj M. Cytodiagnosis of Rosai–Dorfman disease masquerading as lymphoma: a case report with brief review of literature. *J Lab Physicians* 2018;10:460–3 30498323 10.4103/JLP.JLP_69_18 PMC6210841

[46] Gupta K, Bagdi N, Sunitha P, Ghosal N. Isolated intracranial Rosai–Dorfman disease mimicking meningioma in a child: a case report and review of the literature. *Br J Radiol* 2011;84:e138–41 21697409 10.1259/bjr/15772106 PMC3473498

[47] Alwani MM, Elghouche AN, Schueth EA, Campiti VJ, Matt BH, Yekinni AO. Manifestations of pediatric extranodal Rosai Dorfman disease in the head and neck. *Int J Pediatr Otorhinolaryngol* 2020;131:109851 31901484 10.1016/j.ijporl.2019.109851

[48] Chhabra S, Agarwal R, Garg S, Singh H, Singh S. Rosai–Dorfman disease: a case report with extranodal thyroid involvement. *Diagn Cytopathol* 2012;40:447–9 21630484 10.1002/dc.21737

[49] Li S, Yan Z, Jhala N, Jhala D, Li S, Yan Z, et al. Fine needle aspiration diagnosis of Rosai–Dorfman disease in an osteolytic lesion of bone. *Cytojournal* 2010;7:12 20806072 10.4103/1742-6413.65058 PMC2924529

[50] Sengar P, Rao S. Rosai–Dorfman disease and Hodgkin lymphoma synchronously involving the same lymph node: a rare case report with review of literature. *Curr Med Res Pract* 2019;9:189–92

[51] Raslan OA, Schellingerhout D, Fuller GN, Ketonen LM. Rosai–Dorfman disease in neuroradiology: imaging findings in a series of 10 patients. *Am J Roentgenol* 2011;196:W187–93 21257861 10.2214/AJR.10.4778

[52] Gaddey HL, Riegel AM, Bergquist E. Unexplained lymphadenopathy: evaluation and differential diagnosis. *Am Fam Physician* 2016;94:896–903 27929264

[53] La Barge DV Salzman KL, Harnsberger HR, Ginsberg LE, Hamilton BE, Wiggins RH, et al. Sinus histiocytosis with massive lymphadenopathy (Rosai–Dorfman disease): imaging manifestations in the head and neck. *Am J Roentgenol* 2008;191:W299-306 19020219 10.2214/AJR.08.1114

[54] Mahajan S, Nakajima R, Yabe M, Dogan A, Ulaner GA, Yahalom J, et al. Rosai–Dorfman disease – utility of F-18 FDG PET/CT for initial evaluation and follow-up. *Clin Nucl Med* 2020;45:e260 32349088 10.1097/RLU.0000000000003014 PMC8177955

[55] Albano D, Bosio G, Bertagna F. 18F-FDG PET/CT follow-up of Rosai–Dorfman disease. *Clin Nucl Med* 2015;40:e420–2 26053720 10.1097/RLU.0000000000000853

[56] Lee H, King G, Garg K, Pan Z, Tobin J, Robinson WA. Successful treatment of disseminated Rosai–Dorfman disease with siltuximab. *Haematologica* 2018;103:e325 29599203 10.3324/haematol.2018.188441 PMC6029547

[57] Lee C, Choi N, Lee Y, Park JH, Son YI. A case of nasal cavity and laryngeal involvement of Rosai–Dorfman disease. *Ear Nose Throat J* 2024;103:409–12 34841915 10.1177/01455613211054632

[58] Karakuş V, Dere YM, Soysal DE. Long-lasting follow-up with low-dose steroid in an 18-year-old male with Rosai–Dorfman disease. *Case Rep Med* 2020;2020:5727569 32180809 10.1155/2020/5727569 PMC7063216

[59] Chen E, Pavlidakey P, Sami N. Rosai–Dorfman disease successfully treated with thalidomide. *JAAD Case Rep* 2016;2:369–72 27709124 10.1016/j.jdcr.2016.08.006 PMC5043389

[60] Grogan DP, Winston NR. Thalidomide. In: *StatPearls [Internet]*, Volumes 1–9, 4th edn. Treasure Island (FL): StatPearls Publishing, 2023;9:V9-11-V9-17. https://www.ncbi.nlm.nih.gov/books/NBK557706/

[61] Goyal G, Ravindran A, Young JR, Shah MV, Bennani NN, Patnaik MM, et al. Clinicopathological features, treatment approaches, and outcomes in Rosai–Dorfman disease. *Haematologica* 2020;105:348–57 31004029 10.3324/haematol.2019.219626 PMC7012468

[62] Boissiere L, Patey M, Toubas O, Vella-Boucaud J, Perotin-Collard JM, Deslee G, et al. Tracheobronchial involvement of Rosai–Dorfman disease: case report and review of the literature. *Medicine (Baltimore)* 2016;95:e2821 26886634 10.1097/MD.0000000000002821 PMC4998634

[63] Duan HG, Zheng CQ, Wang DH, Ding GQ, Luo JQ, Zang CP, et al. Extranodal sinonasal Rosai–Dorfman disease: a clinical study of 10 cases. *Eur Arch of OtorhinoLaryngol* 2015;272:2313–18 25318688 10.1007/s00405-014-3297-7

[64] Kandukuri R, Phatak S. Evaluation of sinonasal diseases by computed tomography. *J Clin Diagn Res* 2016;10:TC09 10.7860/JCDR/2016/23197.8826 PMC5198426 28050473

[65] Khalili S, Worrall DM, Brooks S, Morris SM, Farquhar D, Newman JG, et al. Endoscopy versus imaging: analysis of surveillance methods in sinonasal malignancy. *Head Neck* 2016;38:1229–33 27142811 10.1002/hed.24413

[66] Workman AD, Palmer JN, Adappa ND. Posttreatment surveillance for sinonasal malignancy. *Curr Opin Otolaryngol Head Neck Surg* 2017;25:86–92 27846019 10.1097/MOO.0000000000000330

